# Semantic interoperability: ontological unpacking of a viral conceptual model

**DOI:** 10.1186/s12859-022-05022-0

**Published:** 2022-11-17

**Authors:** Anna Bernasconi, Giancarlo Guizzardi, Oscar Pastor, Veda C. Storey

**Affiliations:** 1 Department of Electronics, Information and Bioengineering, Politecnico di Milano, Milan, Italy; 2 Conceptual and Cognitive Modeling Research Group, Free University of Bozen-Bolzano, Bolzano, Italy; 3 Services and Cybersecurity Group, University of Twente, Enschede, The Netherlands; 4 PROS Research Center, VRAIN Research Institute, Universitat Politècnica de València, Valencia, Spain; 5 J. Mack Robinson College of Business, Georgia State University, Atlanta, Georgia USA

**Keywords:** Ontological analysis, Conceptual modeling, OntoUML, COVID-19, SARS-CoV-2, Viral genome

## Abstract

**Background:**

Genomics and virology are unquestionably important, but complex, domains being investigated by a large number of scientists. The need to facilitate and support work within these domains requires sharing of databases, although it is often difficult to do so because of the different ways in which data is represented across the databases. To foster semantic interoperability, models are needed that provide a deep understanding and interpretation of the concepts in a domain, so that the data can be consistently interpreted among researchers.

**Results:**

In this research, we propose the use of conceptual models to support semantic interoperability among databases and assess their ontological clarity to support their effective use. This modeling effort is illustrated by its application to the Viral Conceptual Model (VCM) that captures and represents the sequencing of viruses, inspired by the need to understand the genomic aspects of the virus responsible for COVID-19. For achieving semantic clarity on the VCM, we leverage the “ontological unpacking” method, a process of ontological analysis that reveals the ontological foundation of the information that is represented in a conceptual model. This is accomplished by applying the stereotypes of the OntoUML ontology-driven conceptual modeling language.As a result, we propose a new OntoVCM, an ontologically grounded model, based on the initial VCM, but with guaranteed interoperability among the data sources that employ it.

**Conclusions:**

We propose and illustrate how the unpacking of the Viral Conceptual Model resolves several issues related to semantic interoperability, the importance of which is recognized by the “I” in FAIR principles. The research addresses conceptual uncertainty within the domain of SARS-CoV-2 data and knowledge.The method employed provides the basis for further analyses of complex models currently used in life science applications, but lacking ontological grounding, subsequently hindering the interoperability needed for scientists to progress their research.

## Background

Since the breakthrough of Next Generation Sequencing (NGS) technologies over a decade ago [1], enormous amounts of human genome sequences have been produced, supporting important targeted research on cancer, complex diseases, and human identification. In parallel, NGS has addressed infectious disease and microbial research, including viral typing. This application has gained increasing relevance over the last two years, due to the outbreak of the COVID-19 pandemic. Laboratories around the world started sequencing samples extracted from patients with COVID-19, harbouring SARS-CoV-2 viral bio-material, leading to the collection of several millions sequences [2, 3].

Since the early 2000s, efforts have been made to model and understand the human genome, using conceptual modeling to describe genomics databases [4]. Many research efforts have employed conceptual models’ expressive power for explaining biological entities and their interactions in terms of conceptual data structures [5, 6]. With a focus on human genomics, Pastor et al. proposed the Conceptual Schema of Human Genome [7, 8]. Bernasconi et al. further introduced the Genomic Conceptual Model [9] for driving the data integration steps of processed human genomic samples. Conceptual models have also been employed to represent and communicate genome information about alternative, but less complex, species, such as citrus [10] or bacteria [11]. The Viral Conceptual Model (VCM, [12]) was motivated by the scientific interest in representing the genomic aspects of the virus causing COVID-19, so it could be exploited for integrating the heterogeneous data deposited in different databases [13].

Conceptual models have traditionally been employed to capture and represent the main concepts that exist in a domain, using a level of abstraction that is suitable to develop an information system following users’ requirements. In this sense, a conceptual model provides an *information structuring function* for a given application domain. The VCM focuses on such structuring function for the purposes of characterizing viral genomic sequences. The model has already being successfully used for designing several search and visualization systems [14–16] and has been linked to a Phenotype Data Dictionary [17] and a knowledge base of SARS-CoV-2 mutations’ impacts [18, 19]. A broader application and adoption of the VCM within the context of virology research (by both life science domain experts and information systems’ developers), however, requires that a more ontology-oriented approach is embraced, allowing the unambiguous identification of entities in the context of heterogeneous information.

In [20] (and extended here), we argue that a conceptual model should be able to provide *conceptual clarification* and *unambiguous communication* regarding the nature of entities and their connections, which are assumed to exist in a given domain. This is the *ontological function* of a conceptual model. In this sense, the model must strive to represent the exact *intended conceptualization* (that is, the exact set of possible interpretations) of the domain that it is intended to represent. In other words, the model should be explicit and transparent with respect to its *ontological semantics*. Revealing the ontological semantics of an information artifact is a fundamental type of *explanation* for symbolic models (including conceptual models). In Latin languages such as Portuguese, Italian, Spanish, and French, the terms for explanation literally mean “to unfold” (or to unpack). Thus, we use the term *ontological unpacking* to refer to a process of ontological analysis that reveals the *ontological conceptual model* (a conceptual model in its latter function) behind an information structuring conceptual model (the conceptual model in its former function). As a methodological and tool support for this process, we employ OntoUML, an ontology-driven conceptual modeling language whose meta-model complies with the ontological distinctions and axiomatization of the theoretically well-grounded Unified Foundational Ontology (UFO [21, 22]).

The ontological unpacking process enables semantic interoperability of scientific data models [23] according to the well-accepted FAIR principles [24]. It allows adopters to either endorse existing models *as is* or to highlight their missing elements, thus maximizing the added value of their information [25]. In current FAIRification initiatives [26, 27], interoperability is considered to be achieved simply by employing standards, international terminologies, and classifications that unambiguously define the meaning of concepts [28]. Although this may enhance data FAIRness, it does not necessarily guarantee the consistent understanding of concepts from different systems. Rather, models that are transformed by the ontological unpacking procedure can effectively support applications that ensure the FAIR Interoperability principle.

In the following, we provide an overview of the meaning of ontology, ontological unpacking, and semantic interoperability, extending the well-accepted FAIR principles [24] by considering ontological foundations [25], when applied to a Viral Conceptual Model [9]. ‘Method’ describes how OntoUML is used to encapsulate and expand relevant concepts of the initial model to achieve the expanded version of the initial conceptual model, called the *Ontological Viral Conceptual Model (OntoVCM)*. Our results illustrate: i) example applications of the new model for achieving ontological clarification; ii) the use of the model for extracting shared semantics from the data. We finally discuss future challenges.

### Ontology and the “I” of FAIR

Conceptual modeling [29] refers to the adoption of abstraction techniques for representing artifacts and their semantics, associated with databases and software. A correct conceptual modeling practice requires a sound ontological background. Guizzardi [25] suggests the following interpretations of ontology:

(i) “Ontology” (as a discipline) proposes formal methods and theories for clarifying conceptualizations and articulating their representations;
(ii) “ontologies” are constructs capturing the conceptualizations represented within information artifacts. These ontologies may be foundational when they are generic in nature, such as UFO [30], DOLCE [31] or the Basic Foundational Ontology (BFO, [32]).

Ontological unpacking refers to the process which, using Ontology as a discipline, employs the theory of ontological analysis that reveals the ontological conceptual model that represents an information structure. Note, instead, that “domain-specific ontologies” are simply information artifacts capturing a structured (possibly hierarchical) knowledge content within specific fields; OBO Foundry [33] is a relevant collection of such ontologies, including for example the Gene Ontology [34] and the Experimental Factor Ontology [35].

Within the FAIR principles, Interoperability is defined as “the ability of data or tools from non-cooperating resources to integrate or work together with minimal effort” [24]. The most common approach to achieve interoperability in an information artifact is recursive, as it adopts semantic resources that follow FAIR principles themselves [25]. However, research on achieving semantic interoperability on models that are not already based on FAIR principles is lacking. In agreement with [25], we highlight the need for Ontology theory (in the sense of i) for both building ontologies (in the sense of ii) and designing constructs that capture the conceptualizations represented in information artifacts (such as the VCM), transforming them into ontologies, in fact.

**Fig. 1:**
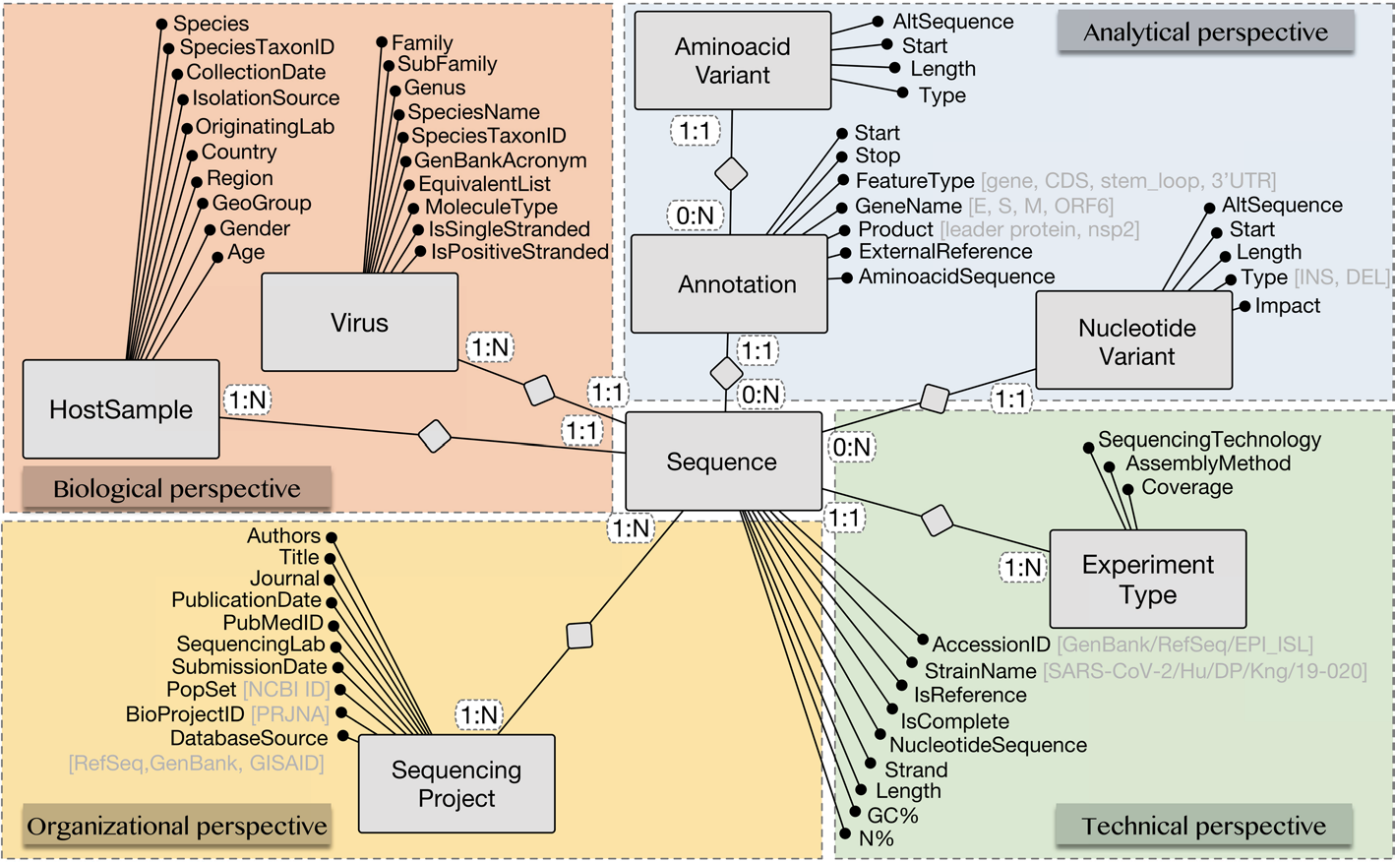
The Viral conceptual model (VCM), from Bernasconi et al. [12]

### The Viral Conceptual Model (VCM)

The Viral Conceptual Model (VCM), shown in Fig. 1, was proposed in [12] as an Entity-Relationship diagram [29] that provides a synthetic and unifying view of the viral sequences metadata universe, with the specific aim to organize the domain and build effective search systems upon such model. It is centered around the notion of a virus genome Sequence and organized into four perspectives, respectively describing the technical process and instruments used for sequencing, the biology of the pathogen organism and the infected host organism, the organization and management behind this process, and the variation of the sequence with respect to its expected behavior. Further details are provided in Additional file 1.

### Related work

**Fig. 2:**
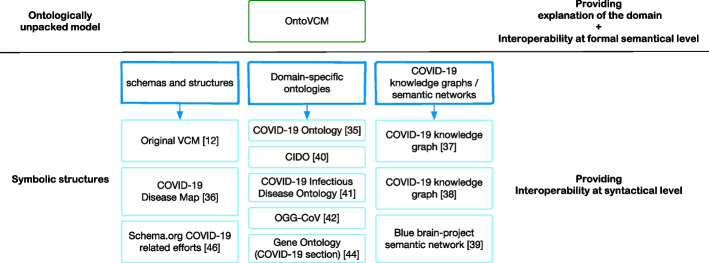
Diagram representing the current proposed solutions for semantic interoperability of COVID-19-related information

Many efforts have proposed ontologies for COVID-19. Specifically, the COVID-19 Ontology [36] is an information structure with two main purposes: i) a template to define context in COVID-19 specific text mining approaches; ii) a structured system of concepts and categories that helps to bring order into the COVID-19 knowledge space. Their focus is on COVID-19 pathophysiology, epidemiology, targets, and medical implications. The COVID-19 Disease Map [37] focuses on the molecular mechanisms of COVID-19 including the generated immune response. Two COVID-19 knowledge graphs [38, 39] are computed as a result of a literature mining processes. The former embraces a very large number of concepts provided as annotations of three mining tools, whereas the latter focuses on cause-and-effect network constructed from scientific literature. A big semantic network, based on entity co-occurrence within literature abstracts, is built within the context of the Blue Brain Project [40] to respond to similar queries. The knowledge representations provided in CIDO [41], the COVID-19 Infectious Disease Ontology [42], and the COVID19 Disease Map [37] capture host-coronavirus interactions mechanisms and their interactions with individual drugs, also including some concepts related to the viral replication process. OGG-CoV [43] is a high-level ontological representation of genes and genomes with several classes/properties for the SARS-CoV-2 structure. The Gene Ontology [44] has a page [45] dedicated to human genes involved in the disease, which is useful to understand how viral proteins interact with the host cells. All such approaches target properties of the disease caused by SARS-CoV-2 and propose information structures without discussing the ontological foundations of their models. Purely syntactical efforts in support of COVID-related data interoperability have been undertaken by initiatives of Google [46] and Schema.org [47, 48] for structuring information and by FAIRsharing [49] for aggregating datasets [50]. See Fig. 2 for a diagramatic representation of the mentioned approaches.

Our approach is complementary both in terms of covered domain and of expressivity. Regarding the first, OntoVCM focuses on describing the infection, sampling, sequencing, and annotations events of SARS-CoV-2 sequences. OntoRepliCov [51] is perhaps the work whose domain is more similar to ours, It shows an initial conceptual framework targeting the translation event during SARS-CoV-2 replication, but is restricted to such mechanical aspects.

With respect to expressivity, unlike purely computational ontologies in limited logical languages such OWL or SWRL (as [51]), OntoVCM is defined as a reference conceptual model [25] represented in an expressive representation language, which can count with full first-order logic capabilities, modality, as well as higher-order types [30, 52]. Besides the lack of expressivity, ontologies directly designed in these computational logic languages are subject to premature optimization issues, often favoring non-functional requirements (such as computational tractability) at the expense of truthfulness to the domain being represented and conceptual clarity [53]. In contrast, thanks to the approach adopted here, from the OntoUML version of OntoVCM, several OWL/SWRL representations can be generated satisfying different sets of non-functional requirements for the same ontology [54, 55].

Despite being coded in OWL/SWRL, some of the ontologies above are conceived with the support of the BFO foundational ontology [36, 41, 42]. BFO is largerly compatible with the UFO ontology adopted here. However, UFO is among the existing foundational ontologies with the most mature theory of relations and relational properties [56, 57], and of higher-order types [58]. There are many aspects of this domain that are inherently relational (for instance, a viral infection requires a virus collective and a host; a virus genetic sequence variant requires a relation to a reference sequence), and many aspects that require the modeling of higher-order types, that is, types whose instances are themselves types (Virus Species, Genomic Assembly Method, Aminoacid Type).

## Method

OntoUML is a language whose meta-model complies with the ontological distinctions and axiomatization of the theoretically well-grounded Unified Foundational Ontology (UFO [21, 22]). Here, we provide a selected subset of the ontological distinctions proposed by UFO, represented by the modeling primitives of OntoUML. The philosophical justifications, formal characterizations and empirical support for the primitives are provided in [30, 56].

**Fig. 3:**
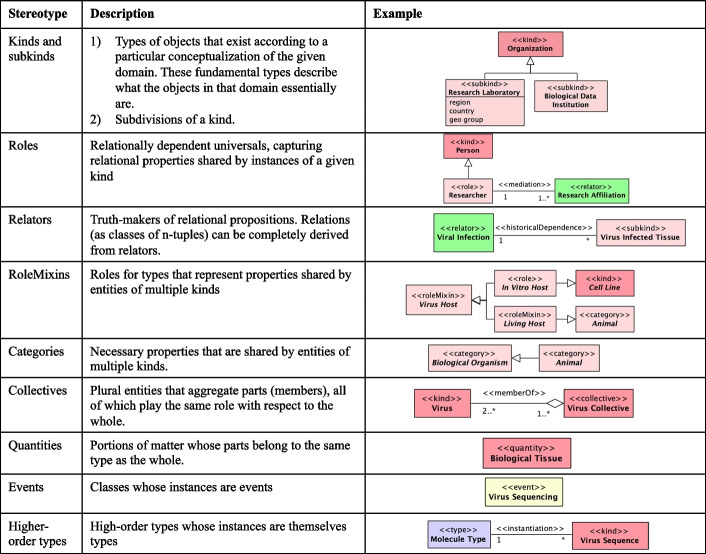
Overview of a part of OntoUML stereotypes, with their description and examples taken from the proposed OntoVCM

The most dominant subcategory is that of *endurants* [30] (as opposed to events or occurrents). Endurants are entities that have essential and accidental properties and, hence, that can change in time. Central to any conceptualization of endurants is a number of object *Kinds*. That is, the genuine fundamental types of objects that exist in this domain. The objects classified by a kind could not possibly exist without being of that specific kind. All objects necessarily belong to exactly one kind and cannot change kinds; Typical examples include Person, Virus, and Organization. There can be other static subdivisions (or subtypes) of a kind, naturally termed *Subkinds*. For example, the kind ‘Organization’ can be specialized in the subkinds ‘Research Laboratory’ or ‘Biological Data Institution’ (first row of Fig. 3). Object kinds and subkinds represent essential properties of objects (also termed rigid or static types [30]).

There are, however, types that represent contingent or accidental properties of objects (termed anti-rigid types [30]). These include *Phases* and *Roles*. Phases represent properties that are intrinsic to entities (for instance, ‘being a puppy’ is being a dog in a particular developmental phase; ‘being a living person’ is being a person who has the intrinsic property of being alive; ‘being available car’ is being a car that is functional and, hence, can be rented). Roles, in contrast, represent properties that entities have in a relational context. For example, ‘being a host organism’ is to bear a number of properties in the scope of viral infection; ‘being a researcher’ is to bear a number of commitments and claims towards a research organization in the scope of a research affiliation (second row of Fig. 3).

Kinds, Subkinds, Phases, and Roles are categories of object *Sortals*. In philosophy, a sortal is a type that provides a uniform principle of identity, persistence, and individuation for its instances [30]. A sortal is either a kind (such as ‘Person’) or a specialization of a kind (such as ‘Adult’, ‘Woman’, ‘Analyist’). Objects can relate to each other via parthood relations forming partonomic structure (for example, a human body is composed of organs and tissues).

*Relators* represent clusters of relational properties that are kept together by a nexus. Moreover, relators (such as enrollments, mandates, affiliations) are full-fledged endurants. In other words, entities that endure in time bearing their own essential and accidental properties and, hence, first-class entities that can change in a qualitative manner while maintaining their identity. Relators are the truth-makers of relational propositions. Relations (as classes of n-tuples) can be completely derived from relators [56]. For instance, it is ‘the affiliation’ (as a complex relator composed of mutual commitments and claims) of the researcher Anna with Politecnico di Milano (PoliMi) that makes true the proposition that “Anna works for PoliMi”. Relators are existentially dependent entities (the affiliation between Anna and PoliMi can only exist if Anna and PoliMi exist) that bind together entities (their relata) by the *mediation* relations – a particular type of *existential dependence* relation [30]. Besides existential dependence, OntoUML allows for endurants to be related by *historical dependence* [57]. While an existential dependence from A to B means that B has to exist in all situations where A exists, a historical dependence from A to B means that, for A to exist, B must have existed before or concomitantly with A. See third row of Fig. 3, where an infected tissue can only exist if a viral infection existed before.

Objects typically participate in relationships (relators) playing certain “roles”: people play the role of ‘Researcher’ in a research affiliation; a person plays the role of ‘Analyst’ in an analyst affiliation. Roles in UFO are relational contingent sortals, since they can only be played by entities of a unique given kind. There are, however, relational and contingent role-like types that can be played by entities of multiple kinds. We call these role-like types that classify entities of multiple kinds *RoleMixins*. An example is the role ‘Customer’ or the host organism for a virus, which could be in vitro or living (fourth row of Fig. 3).

In general, types that represent properties shared by entities of multiple kinds are called *Non-Sortals*. *Categories* are another type of non-sortals in UFO: they represent necessary properties that are shared by entities of multiple kinds; for instance, the category ‘Biological Organism’ represents properties of all living entities that are capable of reacting to stimuli, reproduction, growth, homeostasis, etc., while an ‘Animal’ is a multicellular, eukaryotic biological organism (fifth row of Fig. 3). In contrast to rolemixins, categories are *Relationally Independent* Non-Sortals.

In OntoUML, objects can be *Collectives*; that is, plural entities that aggregate parts (members), all of which play the same role with respect to the whole, or *functional complexes*. In other words, these are entities whose parts (called components) play different functional roles with respect to the whole [30]. As an example a ‘Virus’ is a member of a ‘Virus Collective’ (sixth row of Fig. 3). Objects can also be *Quantities*; that is, portions of matter whose parts belong to the same type as the whole [30], such as a ‘Biological Tissue’ (seventh row of Fig. 3).

Besides endurants, UFO, and hence OntoUML, allow a category of *perdurants* [59, 60]. These are *Events*, which can have their own properties, fall into taxonomies, and be decomposed into parts. However, events only exist in the past and are, thus, immutable in all respects. For this reason, there are two categories of events: event kinds (represented by the stereotype «event») such as ‘Virus Sequencing’ (eigth row of Fig. 3) and event subkinds. The most common relationship between endurants and events is participation, but events can also bring existence (*create*) endurants.

Finally, OntoUML embeds a theory of multi-level modeling and higher-order types [52, 58]. These are represented by the stereotype «type»and are types whose instances are types themselves. A relation of *instantiation* connects individuals to these higher-order types [52], for example ‘Molecule Type’ is the high-order type of a ‘Virus Sequence’ (ninth row of Fig. 3).

## Results

**Fig. 4:**
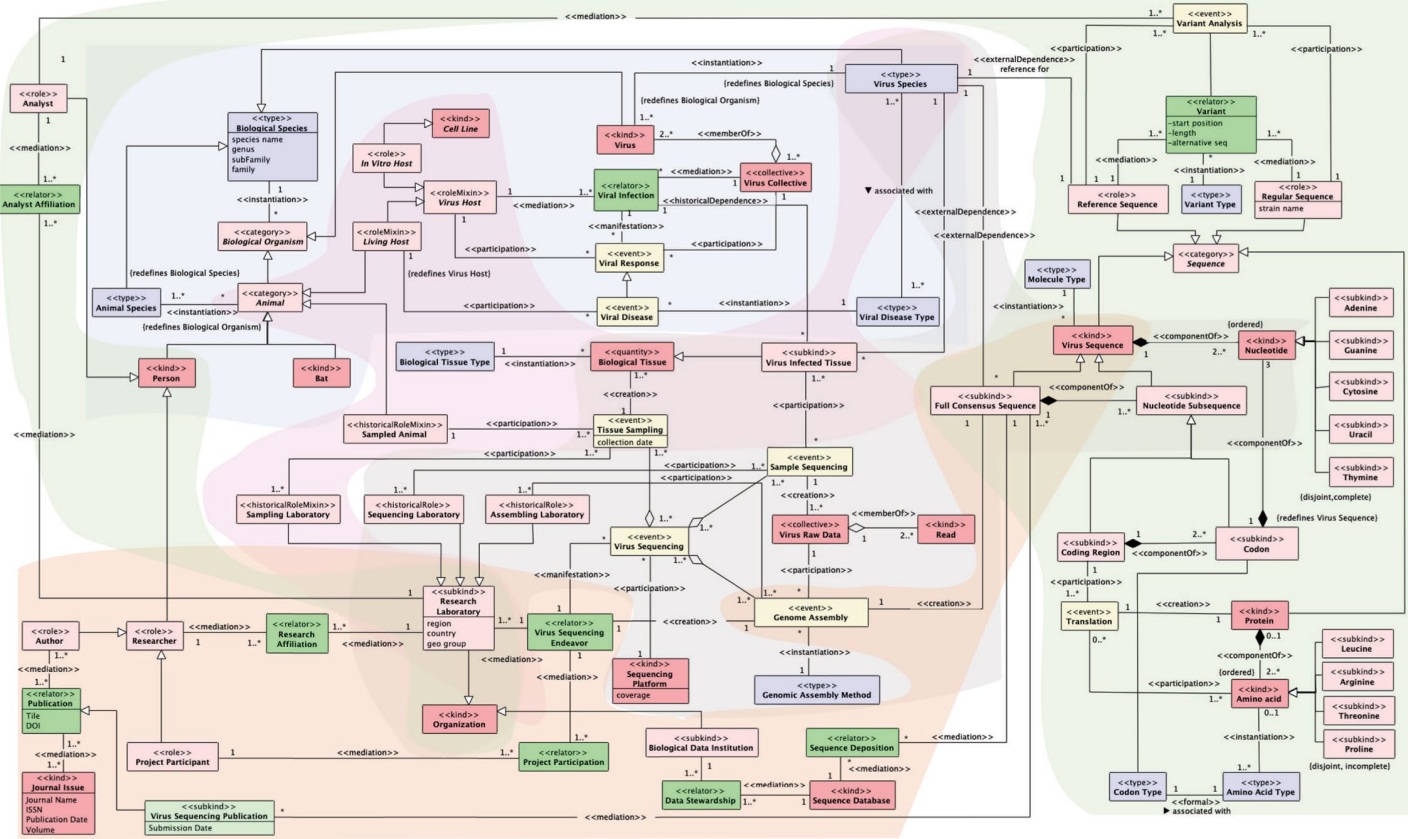
OntoVCM; modules are clustered by background color: Viral Infection (blue), Tissue Sampling (pink), Virus Sequencing (gray), Virus Sequence Publication (orange), Virus Sequence Annotation (green). A more readable version of this figure is available at [61]

We produced an unpacked version of the VCM by reconstructing the original conceptualization underlying VCM using ontological analysis associated with OntoUML. The result of this analysis is captured in a series of modules indicated with different background colors in the complete OntoVCM shown in Fig. 4, respectively capturing aspects regarding Viral Infection, Tissue Sampling, Virus Sequencing, Virus Sequence Publication and Annotation. Following the generally-accepted OntoUML coding scheme, entities’ colors are selected as follows: light red for types whose instances are objects; green for relators; yellow for events; and purple for higher-order types. The details of the ontological unpacking results are reported in Additional file 2, based on [20]. The direct results of such effort correspond to a catalogue of cases explained in the following, where OntoVCM proves effective in: i) clarifying the initial conceptualization and ii) unveiling the data semantics in the SARS-CoV-2 example domain.

### High-level ontological clarification

#### The HostSample case

**Fig. 5:**
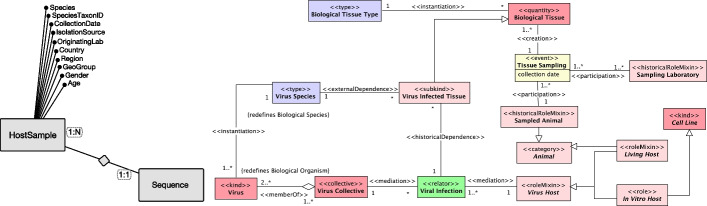
Left: VCM excerpt concerning the biological sample from which the infected tissue is extracted. Right: OntoVCM Tissue Sampling module

VCM includes all information about the host sample in one single entity HostSample, generating a de-normalized structure that conceptually oversights the real-world Tissue Sampling event. This event, which involves actors such as a healthcare worker, the individual who undergoes a swab test, and the facility where the extraction happens, remains under-specified even though it occurs at a precise point in time and space. Figure 5 represents the transformation between the original VCM fragment capturing the aspects regarding the properties of the biological sample of the host organism with the generated sequence and the unpacked repesentation of the Tissue Sampling event involving various actors and the relationships among them.

#### The ExperimentType case

**Fig. 6:**
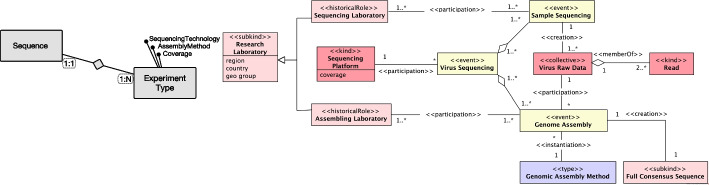
Left: Excerpt of the original VCM technical perspective. Right: OntoVCM Virus Sequencing module

VCM compacts the technological information using the *SequencingTechnology*, *AssemblyMethod* and *Coverage* of the ExperimentType entity, as reported on the left side of Fig. 6. This simplification oversights a more complex representation where a Sequencing Platform kind participates in a Virus Sequencing super-event including a Sample Sequencing event that creates Virus Raw Data collectives (made of Reads) participating in the Genome Assembly event. This excerpt is now more broadly captured by the OntoVCM module on Virus Sequencing (see right-end side of Fig. 6).

#### Describing a process in space and time

Different databases collect SARS-CoV-2 sequence data. Such databases, and similarly the VCM, focus on providing a quick access to data and very minimal descriptions. In doing so, they overlook the complex reality that includes the events of infection, sampling, sequencing, analysis, and submission/publication, as captured instead by the ontological analysis.

Space-wise, sequences are superficially assigned a “location” (*GeoGroup*, *Country*, and *Region* attributes in the HostSample VCM entity), based on the geography of the *OriginatingLab*, whereas the *SequencingLab* is represented in the SequencingProject entity. However, a non better-specified location assigned to a sample could differentially describe: i) the provenance of the infected host (location of the OntoUML Viral Disease event); ii) the laboratory where the virus was collected (Sampling Laboratory historicalRoleMixin); iii) the laboratory where the sample was sequenced (Sequencing Laboratory, OntoUML historicalRole); or iv) the laboratory that took the responsibility of submitting the data to a public database (where the Sequence Deposition event takes place).

Time-wise, the semantics of different registered time points remains ambiguous. For example, the *CollectionDate* of the VCM HostSample and the *SubmissionDate* of the SequencingProject are not always clearly distinguished, even if the two real world events may happen several months apart.

The OntoVCM provides a specification in spatial/temporal terms that supports: i) a correct description of the reality of viral infection, its spreading, and the effects on the moving host (in spatial and temporal terms); ii) disambiguation among different semantics of location and dates assigned to a viral sequence. Note that semantic differences and interpretations of such concepts may strongly impact the understanding and use of the related data within downstream analyses targeted to inspecting variants routes [62], their emergence [63, 64], or their phylogenetic evolution [65].

#### The Translation event

**Fig. 7:**
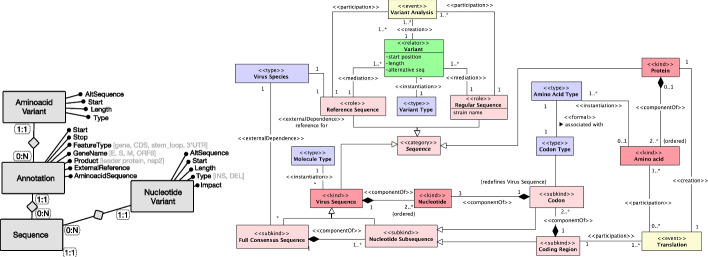
Left: VCM excerpt concerning the sequence with its variants on the nucleotide and amino acid levels. Right: OntoVCM Virus Sequence Annotation module

Public databases and the VCM flatten information regarding very different biological entities (genes, peptides, coding regions, etc.) as shown on the left side of Fig. 7, leading to confusion especially to users unfamiliar with the domain. It is important that nucleotide and amino acid-level mutations are correctly imputed to the belonging genes, proteins, or functional regions of the virus. However, VCM NucleotideVariants are not connected to Annotations, so their meaning within their context (a gene or an untranslated region) is not explicit.

Instead, there is a need to explicitly represent the translation concept as an event as allowed by OntoUML (see right side of Fig. 7). A Virus Sequence instantiates a Molecule Type: here, we restrict ourselves to single-stranded molecule types, based on the focus of VCM. A Virus Sequence is ultimately a sequence of Nucleotides. A Full Consensus Sequence is a Virus Sequence composed of a number of Nucleotide Subsequences, which provides further structure to the Nucleotides composing that Full Consensus Sequences. Nucleotides are of certain well-known types. In DNA they are A, C, G, T; in RNA T is replaced with U. Relevant Nucleotide Subsequences are Codons – sequences of three Nucleotides responsible for coding particular Amino Acid Types – and Coding Regions – aggregations of Codons responsible for coding particular Protein types. A Coding Region participates in Translation events, which produce amino acid sequences; that is, Proteins composed of a particular sequence of Amino Acids (of which 20 subtypes are known). The type of Protein created by a Translation event can be derived from the involved Coding Region. Virus Sequences and Proteins are subkinds of Sequence. A Sequence has two possible roles: Reference Sequence (unique for a Virus Species) and Regular Sequence. These two entities are mediated by the relator Variant, which is of a given Variant Type (such as insertion, deletion, etc.). This relator is created during a Variant Analysis event, to which one reference and one regular sequence participate. Further details can be appreciated in the full ontological analysis reported in Additional file 2.

This new representation enables two important consequences, previously overlooked on the VCM: i) the analysis of nucleotide variation in the context of the specific areas of the sequence to which they belong (with possible different functions); ii) the link between nucleotide variants and their possible mutation at the amino acid level, leading to differentiating synonymous (silent, neutral) and non-synonymous nucleotide (potentially deleterious) mutations.

### Semantics extraction from data

#### Datasets integration

**Fig. 8:**
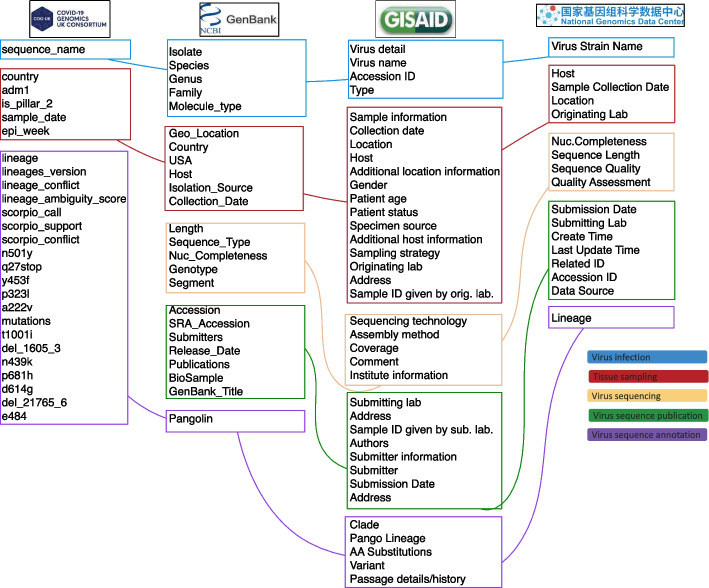
Attributes describing SARS-CoV-2 sequences, after our attempt to assign them to semantic modules described by the OntoVCM (see colored legend)

SARS-CoV-2 data is mostly shared within the deposition database EpiCoV$^{TM}$, hosted by the GISAID Initiative [66], which – as of January 2022 – collected more than 7 million sequences from worldwide laboratories. However, several sequences are still being uploaded to alternative databases: GenBank [67], COG-UK [68], and the Chinese Genome Warehouse [69]. Currently, there does not exist a unifying ontology that supports the seamless integration of such databases, which hold different definitions and formats for representing both metadata and the genomic data itself. Data sources employ very basic data schemata (see Table S1 in Additional file 3), comprising a list of sequences, each connected to many mutations. In turn, mutations can appear in multiple sequences. Metadata describing sequences are flattened and attached as simple lists to each sequence; Fig. 8 shows how the mentioned four relevant data sources cover differently and use heterogenous measures for expressing the information about virus infection, sampling, sequencing, publication and annotation. The VCM has proposed one possible global schema that gathers essential information and provides mappings from each source to offer an integrated interface (with a data warehouse perspective inspired to the Global-as-View approach [70]). However, a higher level ontology is needed so that different semantic interpretations of sources are correctly acquired and – only afterwards – integrated, avoiding the problems highlighted earlier.

#### Entity resolution

A huge number of viral sequences (the same) are submitted to two (or more) different systems, as GISAID and GenBank, or GenBank and COG-UK. The same real-world information is thus input within fields, labelled differently, and handled with different formats; that is, committing to different underlying ontologies. Records that represent sequences derived from a same biological sample processed on a specific point in time and space, with a same sequencing machine and setting configuration, should be reconciled in any integrated database. Failing to do so leads to the computation of incorrect statistics, which are especially dangerous in the context of pandemic-related big data collections [71]. A unifying ontological approach can help address entity resolution problems, as it can be noted in the OntoVCM module on Virus Sequence Publication (see Fig. S1 in Additional file 2), with the OntoUML relator that represents the Sequence Deposition mediating between a Full Consensus Sequence and a Sequence Database. The many-to-many cardinality of its relationship shows the possibility for sequences to be submitted to multiple databases explicitly, thereby capturing the problem of redundant representations of same real-world entities.

#### Location/temporal data notation

Over different databases, locations may be expressed in compact multi-level forms (Continent/Country/Region/Province) where some levels may be optional; the same information is sometimes expressed in different languages (one example is Europe/Italy/Lombardia or Europe/Italy/Lombardy) or input at different levels of the hierarchy ( Europe/Italy/Lombardy/Milano or Europe/Lombardy/Milan). Some sources divide location information into different fields, whose inter-relation is not clear (country=”Italy:Lombardy” and isolate=”SARS-CoV-2/human/ITA/Sars-CoV2-SpikeB_Milan_Oct2019/2019”). Dates are also provided in various formats. Incompleteness often happens at the day level, but can also occur at the month level – many sequences are just assigned the year value ”2020”, which make them useless for data analysis. Such missing information is sometimes expressed with null/empty values; other times it may be filled with fictitious data (such as ‘01’ or ‘Jan’, typically making an incomplete record correspond with the first available date). Moreover, collection and deposition dates are often confused. All these data quality aspects clearly bring negative consequences to data analysis results and studies, especially when they are not made explicit. The ontological unpacking reflects a precise semantics of the domain, enforcing a correct extraction of location/time-related semantics from data (as demonstrated in the above ‘Describing a process in space and time’ section).

#### Virus structure description

The structure of the SARS-CoV-2 virus includes 12 open reading frames (ORFs). Among these, ORF1a and ORF1b are two long overlapping ORFs (accounting for>70% of the total virus length) generating continuous polypeptides, divided into 16 nonstructural proteins (NSPs). The translation of ORF1b is mediated by a -1 frameshift that allows translation to continue beyond the stop codon of ORF1a [72]. Some data sources use the genes notation ORF1a and ORF1b. Others use the proteins notation pp1ab and pp1a, and still others directly use their (contained) non structural proteins notation NSP1–NSP16.

The two most used reference sequences are named NC_045512 [73] within GenBank and hCoV-19/Wuhan/WIV04/2019 [74] within GISAID. They respectively have 29,903 and 29,891 bases and slightly differ also for the representation of proteins. UniProtKB [75] and NCBI Gene [76]) provide structured annotations of its regions and functional domains. However, currently, there does not exist a precise ontological characterization of all the elements of the viral genome, making explicit which terms are synonyms and which are instead connected by more complex relationships.

#### Meaning of variant

The intended meaning of variant (or mutation) is another aspect of interest: a mutation is a deviation with respect to a standard—expected—behavior, which results into a different letter in a string that represents a sequence. The choice of a reference sequence is thus necessary to give context to a variant. When a unique standard is lacking, mutations are expressed (in different databases) using various terms and coordinates. Moreover, substitutions and deletions are either of arbitrary length or reported as only one-base or one-residue strings.

While considering variants, technical limitations of the sequencing process should be taken into account. The experimental practices that attempt to encode biological entities within information structures, in several occasions do not succeed in representing correctly the reality. This can happen for several reasons: the biological material is not sufficient to capture a given phenomenon; the quality of the technical equipment is not adequate; or the coverage of the measurement is too limited. What meaning should be associated with a mutation then? A different letter in a given position could indeed represent a new varied virus or simply an error/lack of measurement in that position.

A correct data representation of the genome cannot ignore these aspects, so several additions to the VCM are needed, as highlighted by the use of OntoUML stereotypes. First, note that several outcomes depend on the results of SARS-CoV-2 variants recognition kits, impacting socio-economic and epidemiological decisions. Thus, making the Virus Sequencing and the Variant Analysis events explicit clarifies a series of problems related to the transformation of a biological phenomenon into the corresponding information structure. We highlight the need of using one unique reference sequence, clarifying the ontological foundation of the virus structure. A Protein is a kind and all the real data instances that refer to a same protein may have different names or slightly different coordinates on the nucleotide sequence. To integrate different sources, it is important that all their annotations map to the same instances of the OntoVCM Protein entity. Similarly, if a Variant expresses a relator between two Sequences – respectively playing the role of Reference Sequence and of Regular Sequence – then data sources instances should map to this representation at the upper ontology level. We conclude that, as different data sources may commit to different “representations of the virus” (that is, different underling ontologies), their results should not be seamlessly aggregated within statistics without prior mapping their ontologies to a shared reference coordinate system.

## Discussion

The progression of research in the life sciences requires the development and use of databases that can be shared amongst scientists and researchers. Before the data can be used, the databases must be integrated, requiring semantic interoperability. Data integration is still challenging, especially for domains where data grows uncontrollably and at very fast rates, as within the SARS-CoV-2 domain. Ultimately, it is essential [77, 78] to share such data within large scientific communities to faciliate efficient processing and analysis from different sources. With the structuring of genomic data based on international standards, data are easier to merge and analyze.

We have argued that adhering to the genomic standards requires a prior shared understanding of the domain. We first showed how the original VCM offers a restricted representation of the reality, hindering the correct understanding of all the processes behind the production and use of viral sequences. We have thus proposed the ontological unpacking as a method to unveil the semantics of a complex domain hidden behind information structures. Designing ontologically unpacked models responds to a general call of the scientific community that requires not only Findable, Accessible, and Reusable datasets, but also Interoperable ones. We interpret interoperability in the sense defended by Guizzardi [25], which requires the use of Formal Ontology, as a discipline, and representation languages based on formal ontological principles, for grounding interoperable artifacts.

The ontological representation of a conceptual information structure, such as the VCM, can also provide the basis from which to investigate further aspects of the virus representation. It uncovers the need for separating the description of the biological entities (concepts) from their representation within information structures (data). For example, in OntoVCM a Full Consensus Sequence is a type of complete Virus Sequence record. Given the initial purpose of VCM, these entities are information/representation entities and not actual chemical structures. In reality, a Full Consensus Sequence is a representation of a virus sequence type that is instantiated by actual complex chemical structures (sequences of nucleotides). An ontological representation makes this distinction explicit. Similarly, the OntoVCM Translation event is not the actual biochemical event involving the real-world counterpart of these entities, but, rather, an information processing event that generates Protein representations from Coding Region representations.

When adopted within clinically-relevant contexts, OntoVCM can be fundamental in allowing a shared understanding of the viral information, especially when this information needs to be joined with characteristics of the infected host. Consider, for instance, a scenario where a hospital with COVID-19 patients is interested in assessing their clinical phenotype, possibly annotating the collected swab tests with additional information regarding the specific SARS-CoV-2 variant and the presence of given mutations with known effects [18]. Tools such as SnpEff [79], CorGAT [80], or the Coronavirus Typing Tool [81] may be used for annotating the viral information, within protected environments such as VirusLab [82]. A correct ontological characterization of concepts is necessary for 1) interpreting all the aspects related to the production of virus sequence, 2) establishing a correct host-virus link, and 3) supporting effective interoperability [17], thereby enabling the design of models that correlate clinical outcomes with the characteristics of the virus.

We presented OntoVCM as a *reference conceptual model* providing conceptual clarification and ontological grounding for a particular perspective on the domain of viral information (infection, sampling, sequencing, annotations and depositing). As such, the model aims at supporting tasks such as domain understanding, unambiguous communication, and meaning negotiation, all of which are essential for semantic interoperability. With the support of the existing OntoUML tool ecosystem [54], from this conceptual model a number of computational implementations can be systematically produced for different implementation platforms ranging from relational databases, to constraint satisfaction visual simulation languages (such as Alloy), to computational logic languages. Note that representations (such as RDF, OWL, OWL-DL, SWRL, etc.) directly enable processes of data annotation [79, 83] and automated reasoning [84, 85]. Reasoning can be extremely powerful within the context of knowledge concerning viral mutations and their impacts, where the most promising directions, based on the taxonomy of Keet et al. [85], are finding gaps in an ontology, discovering new relations, comparing ontologies, using hierarchies of relations, building complex queries interoperable. Following this stream, one can produce different implementations that share the same conceptual worldview but also implementations that are ontologically sound by design. In particular, one of the OWL implementations generated by these tools is directly grounded on gUFO (an expressive OWL implementation of UFO) [86]. Because of it, these implementations can benefit from a general formalization and reasoning support of foundational axioms dealing with events, situations, change, higher-order types, etc. In general, different mappings are required to address different sets of non-functional requirements. Future work is needed to map a number of requirements for different classes of applications that can be based on our model. These classes will then inform our choice of mappings that will be implemented and made available to the research community.

The intersection of conceptual models and ontologies with Machine Learning has been considered in [87]. Specifically, research on knowledge graphs provide important technologies for linked data and ontologies in general [88] and conceptual modeling in particular, including reasoning and querying over contextual data, and rule/axiom mining. OntoVCM will inspire the use of knowledge graphs for representing sequences, their mutations, co-occurrences, and effects; learning techniques will be employed on such structure.

From the standpoint of domain understandability, OntoVCM can be extended by addressing the principles of identity of viruses and their mutations. The original VCM identifies a specific sequence of a virus by means of identifiers of the deposition databases where it was stored and, possibly, by the information on the publication record with which the sequence was first presented. However, mechanisms of identification are missing. What are the identifying characteristics for considering the sequence of a certain species? This is particularly interesting when we allow for modifications; that is, mutations, that can consistently change the functionalities of the virus. In the literature, taxonomies [89] are defined, but not with precise ontological terms. Our ontological representation provides a starting point for precisely defining identifying properties of a virus and its extracted sequence. A definitive characterization of this aspect may also lead to a generalization of the model to completely include all known viruses. It may also be possible to interoperate information between different viruses.

The most relevant limitations to the adoption of the proposed technique are practical. Considerable time is required to perform an ontological analysis; therefore, the joint work of different ontology engineers and scientists must be taken into account. However, the desiderable goal of reaching a shared understanding of a complex domain should justify the effort. Prior knowledge of the OntoUML framework and a background with foundational ontologies and their principles is required to perform a sound ontological analysis and unpacking. However, typically adopters do not need to fully understand the concepts provided in the input models or structures, because the understanding will be assessed and completed while the analysis is conducted.

Moreover, OntoVCM restricts its scope to single-stranded viruses. This was motivated by the need to prioritize a strategy that would include the case of SARS-COV-2. However, since OntoVCM is meant as a general reference model for viral information (under the perspective adopted here), in future work, we will extend our model to include the analysis and modeling of double-stranded viruses.

## Conclusions

Research on the human genome and virology has revealed the need to facilitate the sharing of databases that support such work. In this research, we have shown how conceptual models can support the semantic interoperability needed for sharing among databases and assessed the ontological clarity of these models to support their effective use. This modeling effort was illustrated by an application to the Viral Conceptual Model that captures and represents the sequencing of viruses. Our work was inspired by SARS-CoV-2, the virus responsible for COVID-19. The “ontological unpacking” was accomplished by applying the stereotypes of the OntoUML ontology-driven conceptual modeling language, to propose an ontologically grounded model. OntoVCM is based on the initial model but with guaranteed interoperability among the data sources that employ it. In doing so, we support the desired “I” (interoperability) in FAIR. Further research is needed to apply these modeling efforts to other life science applications.

We have already been applying our method of “ontological unpacking” to other complex domains and, in particular, other sub-domains in the life sciences including a conceptual model of the human genome. The work described in  [90] presents the first results of this initiative with a focus on biological pathways that describes the chemical reactions that explain the different molecular processes in the human body. Such results will be complemented with efforts to address other dimensions of human genomic information including: a structural view (composition of transcribable chromosome elements); a variation view (types of changes that may occur in the genome); a transcription view (process of moving from DNA to RNAs); a proteome view (characterizing proteins structure and properties); and a bibliography and data sources view (identifying relevant information related to sources of valid information, such as KEGG [91] or Reactome [92]). Our ultimate goal is to produce a strongly and precisely connected ontology network for human genome sharing, making available both a system of reference conceptual models and derived implementations with clear indications of the motivating non-functional requirements.

### Supplementary information

**Additional file 1. **The Viral Conceptual Model (VCM) description.

**Additional file 2. **The ontological unpacking analysis of the original VCM to produce the resulting OntoVCM.

**Additional file 3. **SARS-CoV-2 sequence descriptions attributes.

## Data Availability

The schemata have been realized with Visual Paradigm Community Edition (v16.3) and the OntoUML plugin, release 0.5.3 (https://github.com/OntoUML/ontouml-vp-plugin). A full representation of OntoVCM is available at https://tinyurl.com/OntoVCM-complete-figure.

## Abbreviations

CM: Conceptual modeling
FAIR: Findability, accessibility, interoperability, reusability
OntoUML: An ontologically well-founded language for ontology-driven CM; it is built as a UML extension based on UFO
OntoVCM: Ontological viral conceptual model
SARS-CoV-2: Severe acute respiratory syndrome coronavirus 2
UFO: Unified foundational ontology
UML: Unified modeling language
VCM: Viral conceptual model
