# Supplementary material for: Semantic interoperability: ontological unpacking of a viral conceptual model
Source: BMC Bioinformatics. 2022 Nov 17;23(Suppl 11):491. doi: 10.1186/s12859-022-05022-0 (PMC9672571; doi:10.1186/s12859-022-05022-0)
Supplement: Supplementary file 1 — Additional file 1. The Viral Conceptual Model (VCM) description. [file 12859_2022_5022_MOESM1_ESM.pdf]

Additional File 3

Anna Bernasconi [1], Giancarlo Guizzardi [2,3], Oscar Pastor [4], and Veda C. Storey [5]

[1] Dept. of Electronics, Information and Bioengineering, Politecnico di Milano
[2] Faculty of Computer Science, Free University of Bozen-Bolzano
[3] Faculty of Electrical Engineering, Mathematics and Computer Science, University of Twente
[4] PROS Research Center & VRAIN Research Institute, Universidad Politècnica de València
[5] J. Mack Robinson College of Business, Georgia State University

SARS-CoV-2 sequence description attributes

Table S1: Attributes describing SARS-CoV-2 sequences in four data sources

**COG-UK** [1]	**GenBank** [2]	**GISAID** [3]	**NMDC** (https://nmdc.cn/)
sequence_name	Isolate	Virus detail	Virus Strain Name
country	Species	Virus name	Host
adm1	Genus	Accession ID	Sample Collection Date
is_pillar_2	Family	Type	Location
sample_date	Molecule_type	Sample information	Originating Lab
epi_week	Geo_Location	Collection date	Nuc.Completeness
lineage	Country	Location	Sequence Length
lineages_version	USA	Host	Sequence Quality
lineage_conflict	Host	Additional location information	Quality Assessment
lineage_ambiguity_score	Isolation_Source	Gender	Submission Date
scorpio_call	Collection_Date	Patient age	Submitting Lab
scorpio_support	Length	Patient status	Create Time
scorpio_conflict	Sequence_Type	Specimen source	Last Update Time
n501y	Nuc_Completeness	Additional host information	Related ID
q27stop	Genotype	Sampling strategy	Accession ID
y453f	Segment	Originating lab	Data Source
p323l	Accession	Address	Lineage
a222v	SRA_Accession	Sample ID given by the originating laboratory	
mutations	Submitters	Outbreak	
t1001i	Release_Date	Last vaccinated	
del_1605_3	Publications	Treatment	
n439k	BioSample	Sequencing technology	
p681h	GenBank_Title	Assembly method	
d614g	Pangolin	Coverage	
del_21765_6		Comment	
e484		Institute information	
		Submitting lab	
		Address	
		Sample ID given by the submitting laboratory	
		Authors	
		Submitter information	
		Submitter	
		Submission Date	
		Address	
		Clade	
		Pango Lineage	
		AA Substitutions	
		Variant	
		Passage details/history	

References

[1] The COVID-19 Genomics UK (COG-UK) consortium (2020) An integrated national scale SARS-CoV-2 genomic surveillance network. *The Lancet Microbe,*.

[2] Sayers, E. W., Cavanaugh, M., Clark, K., Ostell, J., Pruitt, K. D., and Karsch-Mizrachi, I. (2019) GenBank. *Nucleic acids research,* **47**(D1), D94–D99.

[3] Shu, Y. and McCauley, J. (2017) GISAID: Global initiative on sharing all influenza data–from vision to reality. *Eurosurveillance,* **22**(13).
