# Supplementary material for: Semantic interoperability: ontological unpacking of a viral conceptual model
Source: BMC Bioinformatics. 2022 Nov 17;23(Suppl 11):491. doi: 10.1186/s12859-022-05022-0 (PMC9672571; doi:10.1186/s12859-022-05022-0)
Supplement: Supplementary file 2 — Additional file 2. The ontological unpacking analysis of the original VCM to produce the resulting OntoVCM. [file 12859_2022_5022_MOESM2_ESM.pdf]

Additional File 1

Anna Bernasconi [1], Giancarlo Guizzardi [2,3], Oscar Pastor [4], and Veda C. Storey [5]

[1]Dept. of Electronics, Information and Bioengineering, Politecnico di Milano
[2]Faculty of Computer Science, Free University of Bozen-Bolzano
[3]Faculty of Electrical Engineering, Mathematics and Computer Science, University of Twente
[4] PROS Research Center & VRAIN Research Institute, Universidad Politècnica de València
[5]J. Mack Robinson College of Business, Georgia State University

The Viral Conceptual Model (VCM)

The Viral Conceptual Model (VCM), shown in Figure 1, was proposed in [1] as an Entity-Relationship diagram [2] that provides a synthetic and unifying view of the viral sequences metadata universe, with the specific aim to organize the domain and build effective search systems upon such model. It is organized into four perspectives and centered around the notion of a virus genome SEQUENCE. A viral sequence can be either DNA or RNA. In both cases, sequences are composed of nucleotides; i.e., guanine (G), adenine (A), cytosine (C), and thymine (T) – replaced with uracil (U) in RNA. A sequence is described by strain name, flags for reference and completeness, strand, string length, and percentages of guanine-cytosine (GC) bases and unknown bases. Four perspectives are provided around the central concept, respectively describing the technical process and instruments used for sequencing; the biology of the pathogen organism and the infected host organism; the organization and management behind this process; and the variation of the sequence with respect to its expected behavior.

From a technical perspective, sequences are derived from one experiment of a given type (EXPERIMENTTYPE entity). In these experiments, the biological material is analysed with a platform of a given *SequencingTechnology* (e.g., Illumina Miseq), which allows us to achieve a certain *Coverage*. Then, *AssemblyMethod*s (i.e., a collection of algorithms) are applied to obtain the final sequence.

From the biological perspective, each sequence belongs to a specific VIRUS, which is described by a complex taxonomy flattened into the attributes *SpeciesName* (e.g., severe acute respiratory syndrome coronavirus 2), a list of comparable forms *EquivalentList* (e.g., 2019-nCoV, COVID-19, SARS-CoV-2, SARS2), *Genus* (e.g., Betacoronavirus), *SubFamily* (e.g., Orthocoronavirinae), and *Family* (e.g., Coronaviridae). A virus species corresponds to a specific *MoleculeType* (e.g., genomic RNA, viral cRNA, unassigned DNA), which has either a double or single-stranded structure. Each strand may be positive or negative. A biological tissue is extracted from an organism that has hosted the virus for a certain amount of time. This is represented by the HOSTSAMPLE entity. The host (of given *Age* and *Gender*) also belongs to a *Species*. The sample is extracted on a specific *CollectionDate*, from a host *IsolationSource* (e.g., nasopharyngeal or oropharyngeal swab, lung), at a precise location identified by the quadruple: *OriginatingLab*, *Region*, *Country*, and *GeoGroup*.

From an organizational perspective, SEQUENCINGPROJECT is a project in which a particular sequencing activity is carried out. Each sequence is connected to a number of studies, usually represented by a research publication (with *Authors*, *Title*, *Journal*, *PublicationDate*), possibly uploaded on public repositories with a *PubMedID*. When a study is not available, only the *SequencingLab* and *SubmissionDate* are provided, along with the *DatabaseSource* where the sequence is

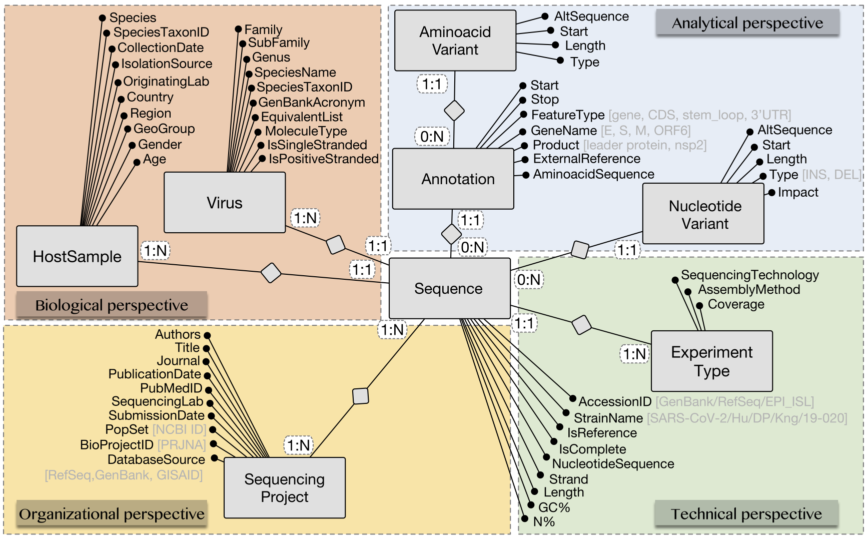

Figure S1: The Viral Conceptual Model (VCM), from Bernasconi et al. [1]

deposited. The study may be optionally linked to a PopSet number and BioProjectID on external NCBI databases [3].

Finally, the analytical perspective addresses the results of secondary analysis of genomic sequences. Annotations include a number of subsequences representing segments (characterized by *Start* and *Stop* coordinates) of the original sequence with a particular *FeatureType* (e.g., gene, peptide, coding DNA region, or untranslated region, molecule patterns such as stem loops and so on); the recognized *GeneName* to which it belongs (e.g., gene "E"); and the *Product* it concurs to produce (e.g., Spike protein, nsp2 protein, RNA-dependent RNA polymerase, membrane glycoprotein, envelope protein...), possibly related to an *ExternalReference* when the protein is present in a separate database. Annotations whose *FeatureType* is coding region (CDS) also have an associated *AminoacidSequence*. The NucleotideVariant entity contains subsequences of the main sequence that differ from the reference sequence of the same virus species. With respect to such reference, they can be defined with a *Start* position coordinate for an arbitrary *Length*, a specific variant *Type* (insertion, deletion, single-nucleotide polymorphism or others), and an alternative sequence of nucleotides (*AltSequence*). Each nucleotide variation may have an *Impact* information, annotating the effect that the variant produces on known genes. A similar role is given to the AminoacidVariant entity, containing subsequences of proteins (i.e., a subset of all annotations) that differ from the reference amino acid sequence of the virus species. These also have a start position, a length, a variant type, and an alternative sequence of amino acid residues.

References

[1] Bernasconi, A., Canakoglu, A., Pinoli, P., and Ceri, S. (2020) Empowering Virus Sequence Research Through Conceptual Modeling. In *International Conference on Conceptual Modeling (ER)* Springer pp. 388–402.

[2] Chen, P. P.-S. (1976) The entity-relationship model—toward a unified view of data. *ACM Transactions on Database Systems (TODS)*, **1**(1), 9–36.

[3] Barrett, T., Clark, K., Gevorgyan, R., Gorelenkov, V., Gribov, E., Karsch-Mizrachi, I., Kimelman, M., Pruitt, K. D., Resenchuk, S., Tatusova, T., et al. (2012) BioProject and BioSample databases at NCBI: facilitating capture and organization of metadata. *Nucleic acids research*, **40**(D1), D57–D63.
