# Supplementary material for: Semantic interoperability: ontological unpacking of a viral conceptual model
Source: BMC Bioinformatics. 2022 Nov 17;23(Suppl 11):491. doi: 10.1186/s12859-022-05022-0 (PMC9672571; doi:10.1186/s12859-022-05022-0)
Supplement: Supplementary file 3 — Additional file 3. SARS-CoV-2 sequence descriptions attributes. [file 12859_2022_5022_MOESM3_ESM.pdf]

Additional File 2

Anna Bernasconi [1], Giancarlo Guizzardi [2,3], Oscar Pastor [4], and Veda C. Storey[5]

[1]Dept. of Electronics, Information and Bioengineering, Politecnico di Milano
[2]Faculty of Computer Science, Free University of Bozen-Bolzano
[3]Faculty of Electrical Engineering, Mathematics and Computer Science, University of Twente
[4] PROS Research Center & VRAIN Research Institute, Universidad Politècnica de València
[5]J. Mack Robinson College of Business, Georgia State University

OntoVCM: ontologically unpacked Viral Conceptual Model

Here, we reconstruct the original conceptualization underlying VCM using ontological analysis associated with OntoUML. The result of this analysis is captured in a series of modules indicated with different background colors in the complete OntoVCM shown in Figure 4 of the main text. Following the OntoUML coding scheme generally accepted by the community, entities' colors are selected as follows: light red for types whose instances are objects; green for relators; yellow for events; and purple for higher-order types.

Virus infection

A Virus is a Biological Organism that can group, forming Virus Collectives. A Viral Infection is a bundle of relational dispositions connecting a Virus Collective and a Virus Host. The latter can be either a Living Host, which is a role mixin played by an Animal that could, be, for example of the kind Bat or Person, or it could be an In Vitro Host, which is a role played by a Cell Line (itself a complex system of cells). The dispositions composing a Viral Infection can be manifested as Viral Disease events in living hosts, whereas cell lines, once infected, clearly show a Viral Response, although this is not considered a disease in full [1]. Viruses are instances of Virus Species, which are associated with Viral Disease Types. All Biological Organisms are instances of Biological Species, which can be characterized by a name, a genus, a sub-family, and a family. Note that, by using the multi-modeling support in UFO/OntoUML, one could go one step further and explicitly capture the *subordination* relations between, for example, species and genus; namely, that a type *is subordinate to* another if, and only if, the instances of the former are specializations of instances of the latter [2]. If a Biological Organism happens to be a Virus then it instantiates a particular type of Biological Species called a Virus Species. Likewise, if a Biological Organism happens to be an Animal, then it instantiates a particular type of Biological Species called an Animal Species. Therefore, in the Virus Infection part of the model of Figure 4 of the main text (blue background), there appears, for example, the instantiation relation between Animal and Animal Species that *redefines* the association ends of the more general relation between Biological Organism and Biological Species. See [3] for an in depth discussion regarding the semantics of redefinition.

Tissue sampling

A Tissue Sampling, central entity of the pink-background module of Figure 4 of the main text is an event occurring in a particular collection date, in which a Sampling Laboratory and a

Sampled Animal participate and in which a Biological Tissue is extracted. A Biological Tissue is a portion of tissue of a given Biological Tissue Type. In the case of an Virus Infected Tissue then that Animal is a Living Host within the scope of a Viral Infection. In other words, a Virus Infected Tissue is *historically dependent* on the existence of a (current or previous) Viral Infection involving that Animal as a Virus Host. Since the Viral Infection is existentially dependent on that particular Animal, it is possible to infer that the Virus Infected Tissue is also *historically dependent* on that Animal. A Virus Infected Tissue is then a sample of viruses of a given Virus Species (the type present in that Viral Infection). Finally, a Sampling Laboratory is a (processual) role played by a Research Laboratory (a specific type of Organization) in a Tissue Sampling event. A Research Laboratory is located in given a country, region, and geo-group (e.g., continent).

Virus sequencing

A Sample Sequencing is an event characterized by a Sequencing Platform, a Sequencing Laboratory, and a Virus Infected Tissue. A Sample Sequencing event creates the collective of Virus Raw Data (comprising many thousands of Reads) from a sampled Virus Infected Tissue. The Virus Raw Data then participates to a Genome Assembly event which is performed by an Assembling Laboratory, and instantiates a particular Genomic Assembly Method. This event produces a Full Consensus Sequence; that is, a complete data record that represents the real virus sequence. A Full Consensus Sequence is composed of a number of Nucleotide Subsequences composed, in turn, of Nucleotides. A Virus Species is associated with molecules of a given Molecule Type. (See the discussion in the 'Virus Sequence Annotation' section). A Virus Sequence (of a virus of that species) instantiates molecules of that type. The events of Tissue Sampling, Sample Sequencing, and Genome Assembly compose a super-event called a Virus Sequencing event, the most crucial part of the gray-background module of Figure 4 of the main text. Notice that, since a Virus Infected Tissue is created by a Tissue Sampling event, we can infer that a Sample Sequencing event must always be temporally preceded by a Tissue Sampling event. Analogously, since a Virus Raw Data collective is created by a Sample Sequencing event, we can infer that a Genome Assembly event must always be temporally preceded by a Sample Sequencing event. Given the transitivity of these temporal precedence relations, we can infer that a Virus Sequencing event is composed of these sub-events that must occur in this particular temporal order.

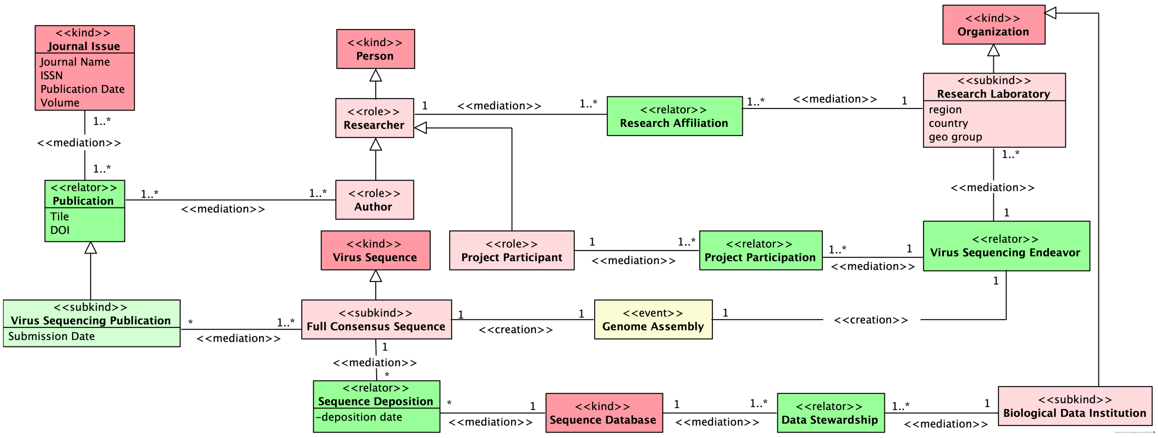

Figure S1: Virus Sequence Publication module of OntoVCM.

Virus sequence publication

As shown in Figure 1 (a zoomed instance of the orange-background module of the full OntoVCM in Figure 4 of the main text, a Full Consensus Sequence is created by a Virus Sequencing event (starting on a tissue collection date and ending with the corresponding Genome Assembly event). A Virus Sequencing is a manifestation of (obligations, rights, capacities) described in a Virus Sequencing Endeavor. A Virus Sequencing Endeavor can have a number of Project Participants, such as Researchers (people affiliated with Research Laboratories). A Full Consensus Sequence has a Sequence Deposition made on Sequence Databases on particular deposition dates. These Sequence Databases are curated and maintained by particular Biological Data Institutions. Here again, we have a bundle of rights and obligations of a particular Biological Data Institution to curate that database following specific protocols and principles. We call this entity a Data Stewardship relator consistent with, for example, the FAIR principles for Data Stewardship [4]. A Full Consensus Sequence can also have a Virus Sequencing Publication, which is a type of Publication (submitted on a submission date). Every Publication has a title and a DOI. It appears in a Journal Issue and is authored by a number of Authors, who are Researchers. Virus Sequencing Publication is a result of the Virus Sequencing Endeavor that the Virus Sequencing manifestation created through the Full Consensus Sequence reported in that publication. A Journal Issue is characterized by the name of the journal to which it belongs (Journal Name), and has an ISSN, Volume and Publication date.

Virus sequence annotation

This module is represented by the green-background part of Figure ??. A Virus Sequence instantiates a Molecule Type. Molecule Types can be either double-stranded or single-stranded; these, in turn, can be either positive-stranded or negative-stranded. Here, we restrict ourselves to single-stranded molecule types because the VCM focuses on single-stranded RNA viruses. A Virus Sequence is ultimately a sequence of Nucleotides. A Full Consensus Sequence is a Virus Sequence composed of a number of Nucleotide Subsequences. A Nucleotide Subsequence, thus, provides further structure to the Nucleotides composing that Full Consensus Sequences. Nucleotides are of certain types. In DNA these are Adenine (A), Cytosine (C), Guanine (G), and Thymine (T), whereas in RNA there is Uracil (U) in place of Thymine. Particular relevant Nucleotide Subsequences are Codons – sequences of three Nucleotides responsible for coding particular Amino Acid Types – and Coding Regions – aggregations of Codons responsible for coding particular Protein types. A Coding Region participates in Translation events, which produce amino acid sequences (i.e., Proteins composed of a particular sequence of Amino Acids). There are 20 known subtypes of Amino Acids of which we only show four examples for space reasons: Leucine, Arginine, Threonine, and Proline. The type of Protein created by a Translation event can be derived from the involved Coding Region. This is because a Coding Region is composed of a particular sequence of Codons, each of which is of a Codon Type, determining a particular Amino Acid Type. So, the Protein created by that event from a particular Coding Region will be composed of a sequence of Amino Acids instantiating exactly those types determined by the Codons composing that Coding Region.

Virus Sequences and Proteins are subkinds of Sequence. A Sequence has two possible roles: Reference Sequence (unique for a Virus Species) and Regular Sequence. These two entities are mediated by the relator Variant, which is of a given Variant Type (e.g., insertion, deletion, etc.). This relator is created during a Variant Analysis event, to which one reference and one regular sequence participate. The event of Variant Analysis is mediated by Analysts, who have an Analyst Affiliation to a Research Laboratory, which is another Organization.

References

[1] Boyd, K. M. (2000) Disease, illness, sickness, health, healing and wholeness: exploring some elusive concepts. *Medical Humanities,* **26**(1), 9–17.

[2] Carvalho, V. A., Almeida, J. P. A., Fonseca, C. M., and Guizzardi, G. (2017) Multi-level ontology-based conceptual modeling. *Data & Knowledge Engineering,* **109**, 3–24.

[3] Costal, D., Gómez, C., and Guizzardi, G. (2011) Formal semantics and ontological analysis for understanding subsetting, specialization and redefinition of associations in UML. In *ER.*

[4] Jacobsen, A., de Miranda Azevedo, R., Juty, N., Batista, D., Coles, S., Cornet, R., Courtot, M., Crosas, M., Dumontier, M., Evelo, C. T., et al. (2020) FAIR Principles: interpretations and implementation considerations. *Data Intelligence,* **1**(1-2), 10–29.
